# Brain‐targeting liposome‐based *APOE2* gene delivery exacerbates soluble amyloid‐β accumulation in *App* ^NL‐G‐F^ mice

**DOI:** 10.1002/alz.088234

**Published:** 2025-01-03

**Authors:** Ni Wang, Tammee M Parsons, Yingxue Ren, Yining Pan, Skylar C Starling, Chinenye Muolokwu, Jagdish Singh, Takahisa Kanekiyo

**Affiliations:** ^1^ Mayo Clinic, Jacksonville, FL USA; ^2^ University of North Florida, Jacksonville, FL USA; ^3^ North Dakota State University, Fargo, ND USA

## Abstract

**Background:**

Alzheimer’s disease (AD) is a progressive neurodegenerative disease and the most prevalent form of late‐life dementia. The ε2 allele of the *APOE* gene encoding apolipoprotein E (*APOE2*) is associated with lower susceptibility to AD among the three genotypes (ε2, ε3, ε4), while *APOE4* is the strongest genetic risk factor for late‐onset AD. APOE plays a critical role in maintaining synaptic plasticity and neuronal function by controlling lipid homeostasis, with *APOE2* having a superior function. Gene therapy that increases *APOE2* levels in the brain has, therefore, emerged as a potential therapeutic strategy to treat AD.

**Method:**

We conjugated PEGylated liposomes with transferrin and Penetratin, a cell‐penetrating peptide, sufficiently deliver chitosan‐APOE2 cDNA plasmid complex into amyloid model *App* ^NL‐G‐F^ knockin mice at 12‐month‐old. Biochemical studies and brain transcriptome analysis were employed to investigate how brain‐targeting liposome‐based *APOE2* gene delivery influences amyloid‐β (Aβ)‐related pathologies in *App* ^NL‐G‐F^ knockin mice.

**Result:**

One month after *APOE2* gene therapy, there was a trend of reduced insoluble Aβ levels in the mouse cortices. Furthermore, in the *App* ^NL‐G‐F^ knockin mice that received the *APOE2* gene therapy, brain transcriptome analysis through RNA‐sequencing identified the upregulation of genes/pathways related to neuronal development. This was supported by increases of *Dlg4* and *Syp* mRNAs coding synaptic proteins in the experimental group. On the other hand, we found that *APOE2* gene delivery increased soluble Aβ levels, including oligomers, as well as exacerbated neurite dystrophy and decreased synaptophysin.

**Conclusion:**

Our results suggest that brain‐targeting liposome‐based *APOE2* gene therapy is potentially beneficial for synaptic formation at the transcriptional level. Forced *APOE2* expressions, however, may exacerbate Aβ toxicity by increasing the dissociation of Aβ oligomers from aggregates in the presence of considerable amyloid burden.

